# An Ishmalite

**Published:** 1888-06-09

**Authors:** Leslie Keith

**Affiliations:** Author of "The Chilcotes," "Uncle Bob's Niece," "East and West,' etc.


### An Ishmaelite.

By Leslie Keith,

Author of "The Chilcotes," "Uncle Bob's Niece," "East and West,' etc.

(Continued from p. 129.)

### Chapter IX.—A New View of Life.

We have all busied ourselves so much over the East-end and its woes, that the claims of the North and the South on our compassion have been somewhat overlooked, and yet in both quarters there is a great deal to interest the visitor, and much honest and brave work accomplished, as well as much straitness and privation patiently borne.

It was on the Surrey side of the river—that hive of industry—that Arthur found the refuge Fred demanded. It is mostly the poor, taken collectively, who live there; but the poor, taken individually, are of all conditions, shades, and degrees, just as are the rich.

Mr. Proudie, for instance, who, with the acumen of his race, knew the advantage of having two strings to your bow, and successfully wedded the diverse businesses of an eating-house and a stationer's shop, held himself and justly—high above the vendor of hot potatoes and chestnuts, who drove a rival trade on the pavement over the way; and yet to the eyes of Mayfair and Belgravia the distinction would doubtless have been invisible. Very few of those who live in the London of pleasure know anything of this London of work, where a hum ascends all day long from the wharves, the wool-stores, the tan works; where sails and rope, brushes, glue, and vellum, and many more useful things are made.

It sufficed for Fred's purpose that no wanderer from the West—the forbidden land for him—was ever likely to discover the stationer's assistant in the little shop in Gillespie Street, Bermondsey.

"I knew Proudie down in Scotland," Arthur was saying, as he led his cousin through this unknown region. "He was butler to the Grahams, my mother's cousins at Spens, where I spent a summer as a little chap, you remember. I ran across him a year or two ago in the oddest way, and haven't lost sight of him since. He has been in London for twenty years, and has done pretty well, I believe, with a kind of restaurant for working men. He's a bit of a character," Arthur went on, trying vainly to give the talk a lightness he did not feel.

"An eating-house? I'm afraid I'll make but a clumsy waiter——"

"It's for the stationery business he wants help," said Arthur hastily. Then he stopped suddenly in the middle of the narrrow, crowded street.

"Fred," he said eagerly, "if you don't like it, remember you're not bound to go in for it. You are as free as I am."

"I will tell you if I don't like it"—Fred smiled sombrely; "but my choice is scarcely as unlimited as yours."

They had fallen upon the busiest hour of the day for their arrival—the mid-hour, when all the great factories and works let out their hands, and the little restaurant was doing a very brisk trade.

Mr. Proudie was himself helping his customers and meeting the demands for pea-soup, slabs of cold pie, hot pork and potatoes, with a cheerful alacrity. He was a fat little man, with a face like a winter apple, lit by a pair of twinkling blue eyes. These spied out the two young men in an instant, and shot a quick glance at Fred, who felt himself reddening under it.

When he had duly delivered the portions he carried—which he did much as if he were dealing with a pack of cards—he wiped his hands on the corner of his white apron, and came over to them.

Arthur shook hands with him.

"Could you take a seat, Mr. Eastgate, sir, for a minute, and when we're slacker, we'll have our bit talk?"

"This is my cousin, Proudie," said Arthur, "of whom I spoke to you."

The Scotchman wiped his hand once more, and held it out to Fred, who hung his head as he felt that honest grasp. If Proudie had ordered him to the door, he could not have humbled him as he did by this frank exhibition of good-will.

"I say, Proudie," called out Arthur, as the bustling master was hurrying away, 'send us something to eat—will you? when you've time—a chop, or anything that you have ready—it does not matter; and something to wash it down with."

"You'll have a chop in five minutes, sir."

He found them a quiet corner and a little table, where they could eat and watch what was going on around them. Fred sat in a gloomy silence. His hands were rough, too, though his coat was that of a gentleman. "If those good fellows, shovelling down their food with their knives, knew whence I had come, is there one of them that wouldn't shun my company?" he thought. "I ought to be waiting on them; my day of being served is over."

Arthur did his best to keep up his own spirits and his companion's. He was secretly elated by the hope that he would vanquish yet; when Fred had tried the life, and had found out how different were its conditions from those to which he had been used—Arthur chose to ignore the prison episode—he would listen to reason and yield in his turn.

When the customers had satisfied their healthy appetites, and the steamy air of the room had gathered a little clearness from the lessening relays of food and of diners, the master of the shop came over to the corner where the cousins sat.

"We're slacker a bit now, till tea-time, gentlemen," he said. "The men are sharp-set on their victuals in the middle of the day."

"You are doing a thriving trade, Proudie, and I hope it is filling your pockets."

"Pretty well, sir; what with this and the stationery business, which the wife and the lassies look after, we manage to rub along."

"It is for the other shop you wanted help?" said Arthur.

"Yes, sir; the women-folks can manage what there is of it. There's my girl Barbara, as active and eydent a lass as steps, can keep the books as well as any man; and Delia has as pretty a way as you could wish of setting out the stock and keeping it tidy; but I was thinking of starting a circulating library, and that's a bit beyond them——"

"Would that pay down here?"

"I think it would, sir, if they had the right stuff. There's readers enough, but the trash they read is just fair awful."

"You want to give them the best sort of food for their minds, as you do for their bodies?"

"Something in that way, sir. I can tackle their bodies," said the little man, with twinkling eyes, "and give them good broth and beef that puts some smeddum into them; but to choose what they're to put into their heads is more than I'm fit for. It's work for a gentleman, that is, Mr. Arthur, and a man of eddication and book-learning."

"If it is the charge of books, and giving them out, and recommending them, I think I could do that," said Fred, speaking for the first time.

The end of it all was that they were invited to accompany the owner to the shop in question, and view the ground for themselves. Arthur passed his arm within his cousin's, as if to give him courage.

It was curious to see how the younger stood by and protected the elder—the grey and haggard man who was so much older than his years. But if Arthur harboured any fears of affront for Fred, he might have relinquished them there and then. Never from the first moment to the last of their association as master and assistant, did Proudie hint by so much as a look that he knew Fred's story. He kept it sacredly from his wife and children; he locked it in his breast. Could any gentleman have shown a finer tact—a truer charity?

The shops adjoined each other, and the cheerful odour of cooking invaded both alike. The family occupied the rooms above in the tall, narrow building. The stationery department was entered by a pair of steep steps, a bell attached to the door announcing the approach of a customer with a sharp jangle. When Proudie and his guests entered it, a young girl, seated behind the counter, was the only occupant. Apparently, custom was not so brisk here as in the neighbouring premises.

Fred looked about him with a faint curiosity. The outer shop was small, low-browed, and dark. There were paper and writing materials of the cheaper order, and a slender fancy stock in the shape of horn-handled pen-knives, sealing-wax, and balls of twine, neatly arranged under a glass case. High up on a shelf above the girl's head was the nucleus of the coming library, in a row of dingy, dilapidated volumes.

The girl's face attract d Fred, too. It was not in the least pretty, its only charm being the broad brow, from which the brown hair was smoothly brushed; but it had a curious look of quiet strength. She had not inherited any of her features from her father, and yet her grey eyes had caught their quick watchfulness from him.

"Well, Barbara, my lass," he said kindly, "have ye done well the day?"

"Not very well, father; but Monday is never a busy day. Do you want mother? She is up-stairs."

"Aye, aye, we'll go up presently. Hand down the books, lass; the gentlemen have come to look at them."

"Let me help you," said Arthur, springing up on the counter.

"I'm afraid they're very dusty," said the girl, whom her father called Barbara.

The dust indeed was eloquent of neglect. It was a queer collection, and, as Mr. Proudie modestly explained, had been chiefly selected for its cheapness from that morgue of dead literature—the street stall.

"But there's nothing bad, so far as I could make out," he said, anxiously; and there's some good sound theology, as my sister Betsy can answer for."

"Sound enough, I dare say; but a little tough in the digestion—unlike your capital steaks," said Arthur, with a smile for the girl looking gravely up at him. "Have you read them?" he asked.

She shook her head. "I tried, but I could not understand them."

"Then they must be pretty bad," said Proudie, ruefully; "for I never knew the book you stuck at yet, my girl."

The inspection over, they followed their leader to the upper quarters, Barbara remaining below in charge of the shop. The narrow stair was so dark that their dazzled eyes could not for a moment take in the aspect of the parlour into which they were ushered by the bustling Proudie. Presently it discovered itself to be a long, low-ceiled room, occupying the space of both shops beneath it. The little windows were all open to admit the hot, languid air, but the unsightly vision of crowding roofs and chimneys was hidden by a neatly-drawn screen of muslin. There were other evidences of deft womanly skill in the long shabby apartment, which Arthur put down to the grave Barbara, with the broad brow, waiting below to sell a pennyworth of writing-paper, or a copy of *The Chronicle* at half-price to some late customer. He put them down to her, till his eyes lit on a young girl crouching by the most distant window, who sprang up at her father's entrance. When Delia had been mentioned, Arthur had smiled to himself. A girl who bore so fanciful a prefix to the unbeautiful name of Proudie, must needs, by all the laws of luck, be as plain as a Betsy or a Susan. But this Delia, if Delia she were, was charming enough to be the inspiration of any poet.

How came any one of so fair and blue-eyed, so innocent a prettiness, to be here? She dazzled the young man, and seemed to illuminate the dark, shabby room with her looks, as she stood, blushing and wide-eyed, staring at the strangers. But Arthur was not long allowed to wonder. There were two others in the room besides the girl who stood afar off and never once took any share in the talk that followed.

"Wife," said Proudie, in his loud, cheerful tones, "here's Mr. Arthur Eastgate and his cousin, Mr. Eastgate, come to see us."

A fat, smiling woman got up laboriously out of a low chair, where she had been sitting with her hands folded on her knees in an apparently contented idleness.

"And glad I am to see Mr. Harthur again," she said, in a soothing, lazy voice; "and to be sure, sir, you 'ave growed as I wouldn't a'most have known you."

"I've had some time, Mrs. Proudie, since the days when you and I consoled each other for our exile from London. My consolation took the shape of jam tarts, I remember."

"Have, not 'ave," a shrill voice broke in on this remark.

Arthur turned round, rather startled, to meet the gaze of an elderly woman of a rather grim, hard-featured aspect.

"It's Betsy Proudie, my sister, who lives with us," said the brother, making the introduction casually. Evidently Betsy's odd way of asserting herself had ceased to have the novelty of a surprise for him. The young men, understanding that hand-shaking was a part of the etiquette of Bermondsey, rose and solemnly performed this ceremony.

Miss Betsy gave Arthur a bony knuckle to press, but she looked at him with something between rebuke and angry disapproval, for which he vainly sought a reason in his conscience.

The talk after this had that spasmodic uncertainty that talk has where half of the company is unused to the social habit. Arthur felt himself desperately plunging back on those old times when he had been little, and the good-natured English housekeeper had comforted his woes with jam. Fred sat silent and constrained through it all.

Presently the young girl, who had been diligently hanging over some sewing, put it down, and slipped from the room. She fled down the narrow stairs, and took refuge behind the counter with her sister. A customer was being served at the moment, and she waited in silence while the purchase was being selected, secured in paper and string, and payment duly made, but almost before the little door had shut behind him, she whispered eagerly——

"Barbara, is he—is he a prince, do you think?"

Barbara turned her clear grey eyes to the pretty, flushed, excited face.

"A prince? No; but just as far removed from us as if he were."

"But—I thought he was coming to be papa's assistant——"

"Not he—the other one, perhaps; though why he should come I do not know. He is unlike us, too."

"Oh, the other one—he looks fierce. I do not like him so well; and he is old——"

"We have nothing to do with him," said the steady Barbara. "See, child, you have tangled that ball of twine——"

"Well, I can straighten it again," said Delia, with a little pout. "But we shall have to do with him if he comes here to serve," she added. "You will have to help him. Shall you like it, Barbara?"

"I cannot say, Del."

Meanwhile Arthur was taking a farewell of his cousin in the small bed-room where Proudie had considerately left him—for the Scotchman was a shopkeeper after the patriarchal manner, and kept his assistants under his own roof. It was a poor little place, but it was scrupulously clean and neat.

"If you had occupied a Dartmoor cell for five years, you would be less fastidious," said Fred, with sad bitterness. "Leave me here awhile, my boy; it is a better refuge than I had any right to hope for. If—if I find myself weaker than I thought, I will send for you."

"I will come without your sending," said Arthur, wringing his cousin's hand, as he went away.

He went down-stairs without any farewells; his heart was very full, and he was so busy with his inward grief and pain, that he walked out of the shop without so much as seeing the girls standing behind the counter, who followed him with silent looks.

*(To be continued.)*

